# A novel Dynamic Body Weight Support overground co-walker enabling variable unloading ratio and Motion Tracking

**DOI:** 10.3389/fnins.2023.1188776

**Published:** 2023-06-08

**Authors:** Xiaoqian Zhang, Peng Shang, Bing Li

**Affiliations:** ^1^Center of Neuroengineering, Shenzhen Institute of Advanced Technology, Chinese Academy of Sciences, Shenzhen, China; ^2^Shenzhen Jieshui Medical Technology Co., Ltd., Shenzhen, China; ^3^Department of Spinal Surgery, Liuzhou Worker's Hospital, Liuzhou, China

**Keywords:** Dynamic Body Weight Support, Motion Tracking, gait training, gait phase detection, overground walk training

## Abstract

Dynamic Body Weight Support (BWS) systems have gained attention in recent years for their potential in gait training. However, maintaining a natural gait and vertical unloading have been less explored. In our previous work, we developed a body Motion Tracking (MT) walker that can move with patients. In this study, we introduce a novel Motion Tracking Variable Body Weight Support (MTVBWS) system for overground walkers. This system utilizes Center of Mass (COM) tracking and gait phase detection to not only dynamically support the user's body weight in the vertical direction but also to facilitate movement in all directions. The system achieves this horizontal omnidirectional movement by employing active Mecanum wheels, guided by COM recognition. The validation experiments were implemented with the MT mode, passive mode, and BWS mode in “static,” “fixed unloading ratio (FUR),” and “variable unloading ratio (VUR)” settings with unloading force of 20 and 30%. The result shows that, compared to other modes, the proposed system in the MTVBWS mode can reduce the dragging effect in the horizontal plane caused by the walker. Moreover, the unloading force can be adjusted automatically to minimize the fluctuations in the force experienced by each lower limb during the rehabilitation walking training process. In comparison to natural walk, this mode presents smaller force fluctuations for each lower limb.

## 1. Introduction

Gait training is an important and effective approach for the treatment of lower limb mobility disorders in mid- to late-stroke, enabling patients to achieve the most natural gait and vertical unloading to the greatest extent possible, where patients usually require repetitive, task-oriented, targeted training programs based on lower limb motor levels (Dong et al., 2021). Patients frequently cannot train independently because of insufficient lower limb strength, and they may require manual assistance or interventions from other BWS systems. Relevant clinical reports suggest that lower limb rehabilitation devices can enhance training effects for such mobility disorders, mainly in terms of BWS, stability, and real-time BWS force adjustments (Moreno et al., 2016). The usual lower limb rehabilitation assessment criteria focus on three areas: muscle activation, BWS capacity, and body balance (Van Thuc et al., 2015), and thus require providing patients with movement flexibility in lower limb training. The appropriate BWS measures can enable them to support themselves with residual strength and to stimulate autonomous maintenance of body balance (Morone et al., 2017; Straudi et al., 2017).

The partial BWS treadmill based on a flexible winch has been proposed to solve the problem of the user's failure to walk on his own strength and to reduce the workload of the healthcare service providers (Wang et al., 2020; Li et al., 2021). The patient is generally fixed to a fixed frame by a flexible cable and walks on a designated slow walking machine, which can adjust the BWS ratio and walking speed according to the walking ability of the lower limbs (Bernhardt et al., 2005; Kwak et al., 2017). However, this type of assistive device utilizes flexible cables to secure users, yet the device cannot follow users in all directions. Consequently, during walking training, the cables exert pulling forces on the users. Under the influence of the cables' elasticity, users are forced to swing unnaturally, resulting in atypical gait patterns and an inability to stimulate the patient's autonomy in maintaining balance (Plooij et al., 2018), while the fixed BWS ratio will also lead to instability during the training process due to the up and down movement of the body during walking (Lu et al., 2013). Although the following research work made it possible to dynamically adjust the BWS ratio according to the load of the flexibles and maintain the BWS ratio as stable as possible, the problem of forced movements such as swaying of the patient's body remained unsolved and the rehabilitation effect of the lower limbs was limited due to the passive and stretchable nature of the flexibles (Seo and Lee, 2009).

To overcome these problems, in recent years, dynamic tracking weight-reducing walkers have been proposed, replacing the flexible cable system with a fixture system (harness) that can actively follow the patient's body in multidimensional directions on the slow-speed treadmill, such as an MT lower limb walking training system fixed at the waist developed by Van Thuc et al. The spatial position of the patient's COM is collected and calculated by multiple force sensors and follows the patient's body movement accordingly (Van Thuc et al., 2015). Li et al. developed a machine learning-based waist-fixed multidimensional following lower limb training system, which is fixed to the patient's lower limb by Inertial Measurement Units (IMUs), calculates the position of the patient's COM in real-time, and adopts a corresponding BWS strategy by analyzing the characteristics of the healthy side and the disabled side of the lower limb (Li et al., 2021). Also, Banala et al. developed a Variable Body Weight Support (VBWS) walking training system based on the combination of a treadmill and lower limb exoskeleton. This system assists walking by compensating for the patient's lower limb hip and knee joint moments, leading to a gait with improved kinematic and spatiotemporal characteristics, such as stride length, cadence, and joint angle trajectories, which more closely resemble those observed in the neurotypical population (Banala et al., 2009). Simultaneously, the application of the plantar pressure distribution system allows the BWS system to more quickly and accurately analyze the acquisition of COM (Di et al., 2016), and the above demonstrated through experiments that in the mode of dynamically following the patient's COM, the BWS effort is more stable and the gait curve is more aligned with the normal human walking curve.

Although a large number of experiments have demonstrated that a walking machine-based MT BWS lower limb rehabilitation system does provide some degree of lower limb rehabilitation for the patients (Han et al., 2014; Martins et al., 2015), there is a growing body of scientific work demonstrating that direct ground contact rehabilitation can still further improve the efficiency of gait training (Uegami et al., 2019), due to the fact that patients can walk freely based on their autonomous consciousness, rather than being constrained by the unidirectional motion of the walker (Aoyagi et al., 2007). Meanwhile, the ZeroG system has been proposed, which can facilitate on-ground gait training through a BWS system mounted onto a trolley that rides along a horizontal guide rail (Nef et al., 2009). This approach minimizes the horizontal forces experienced by patients, thereby achieving vertical unloading. In addition, clinical studies have shown that in the field of neurorehabilitation, free trajectory walking with BWS assistance provided by related rehabilitation walking devices can maximally activate leg muscles, thereby improving kinematic variability and user involvement in active walking (Aurich-Schuler et al., 2017). Moreover, the movable BWS walkers based on this scheme can be used in more scenarios such as home and community (Tan et al., 2012; Sun et al., 2019), enabling more application possibilities and enhancing the user experience at the same time. For example, Dong et al. proposed an active BWS movable frame, which can achieve VBWS under relatively free walking conditions by calculating the position of the body's COM and ground contact state through the feedback of the plantar pressure system, and then projecting the gait phase and dynamically adjusting the BWS ratio of the left and right lower limbs (Dong et al., 2021). Another example is the traction-based VBWS ground walking walker system proposed by Tan et al. which makes the BWS direction almost vertical by adjusting the BWS orientation, thus minimizing the error state of horizontal drag (Tan et al., 2012). However, because the walker itself is heavy due to the installation of multiple sensors and actuators, it requires the patient to rely on body tugging during training, which makes it difficult for the already underpowered lower limbs to complete the training maneuvers (Villa-Parra et al., 2020).

In this study, we present an over-ground mobility aid system that combines the benefits of both omnidirectional horizontal MT and VUR-VBWS capabilities. This system can dynamically apply a doubled BWS ratio during the swing phase of gait compared to other gait phases, effectively reducing the fluctuations in the BWS force experienced by each lower limb. This system integrates the MT of the patient's COM to reduce the drag effect provided by the slow treadmill-based VBWS system and the benefits of over-ground BWS walkers for ground walking rehabilitation training. The design and implementation of the MTVBWS system adopt different methods from those previously mentioned to achieve the desired training outcomes. In the following sections, we will elaborate on the novel system design principles and implementations.

**Figure 1:**
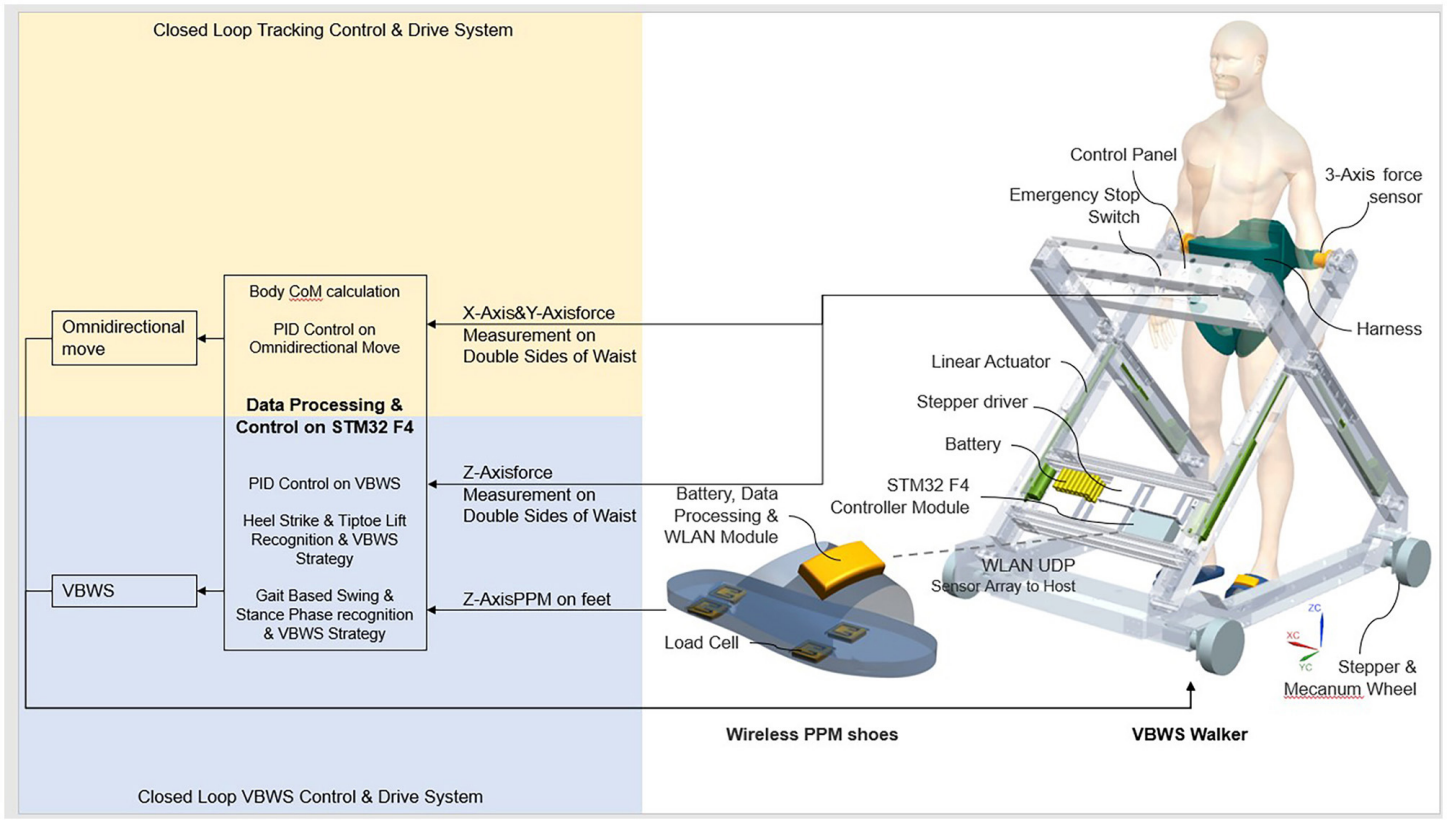
Overall structure and control system of MTVBWS walker.

Figure 1 shows the main components of the system, which include a planar body motion capture system and a VBWS system in the vertical direction. We have enhanced the horizontal motion by incorporating 3D force sensors and a four-wheel drive mechanism based on Macanum wheels, allowing the walker to move omnidirectionally in the horizontal plane based on the motion capture data. The BWS system identifies gait phases and provides different BWS ratios to maintain stability. By monitoring pressure sensor data in the plantar and waist areas, the system distinguishes between the stance and swing phases, subsequently providing different BWS ratios while maintaining minimal BWS force fluctuations.

In this article, Section 2 introduces the architecture of the BWS and MT system, which includes the force-sensing acquisition system, the horizontal omnidirectional motion mechanism, and the zonal BWS mechanism. In Section 3, we focus on the operation of the control system, including the logic behind the execution decision, the closed-loop Proportional-Integral-Derivative (PID) feedback system, and how the system dynamically adjusts the MT and VBWS algorism to minimize drag effect and fluctuations in BWS ratio during gait training. We validate the effectiveness of the system through a series of real-time gait experiments by comparing the performance of our proposed assistive walker in variable BWS mode to its performance in the static body weight support (SBWS) mode, and some performance data related to safety were tested.

Overall, this system allows users to engage in over-ground gait rehabilitation training with minimal drag burden from the walker itself and relatively stable BWS forces. An emergency stop button is deployed on the device, providing an emergency power-off braking function to prevent accidents such as falls. Altogether, this presents a promising solution for lower limb rehabilitation training.

## 2. Materials and methods

### 2.1. Architecture of MTVBWS walker

#### 2.1.1. Force-sensing acquisition module

**Figure 2:**
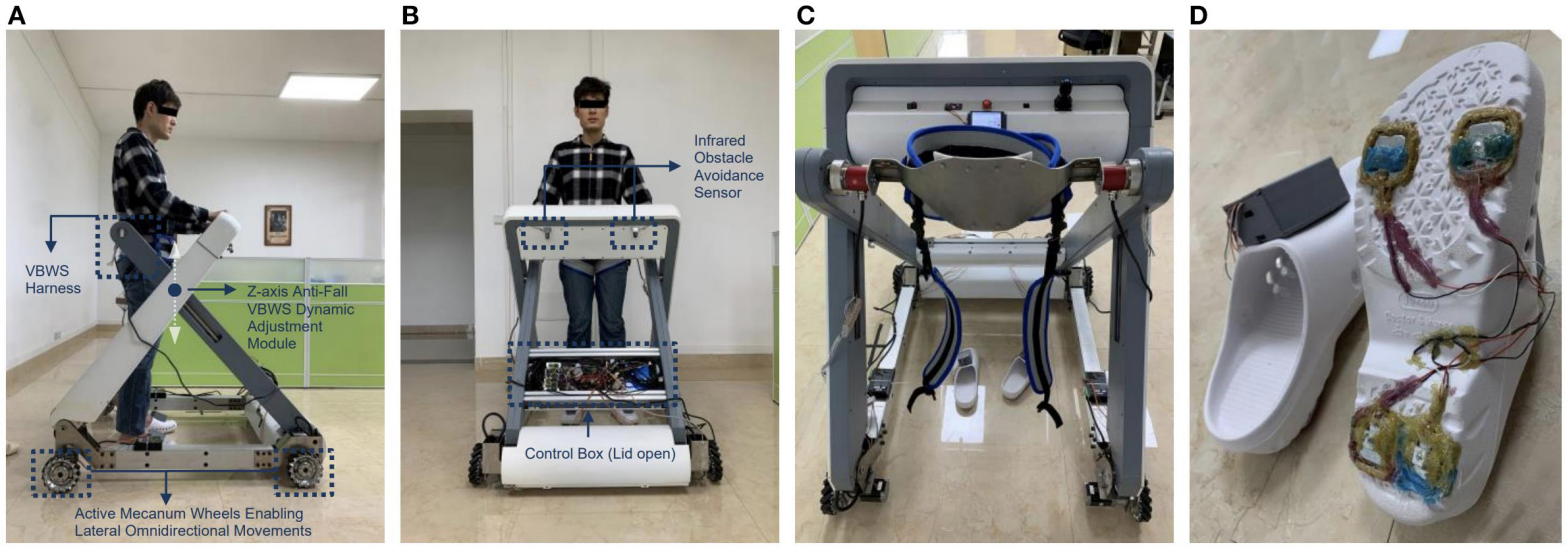
The build of proposed MTVBWS walker system **(A)** Macnum wheels, drive units, and lifting mechanism (side view); **(B)** Control, wireless transmission, and drive system (front view); **(C)** Waist and crotch fixation strap and 3D force sensor; **(D)** Plantar pressure distribution acquisition shoe.

When using the lower limb gait training system, typically there are two areas of contact between the patient and the outside, including the feet to the ground and the waist to the equipment. These areas of contact correspond to the gait force and the weight-bearing force, respectively. The pressure data obtained from these two areas are crucial for evaluating the patient's gait ability, fall prevention, and determining the appropriate BWS strategy for the device. To this end, we have developed a specialized plantar pressure distribution acquisition shoe and a waist and crotch pressure acquisition module, as shown in Figures 2C, D.

**Figure 3:**
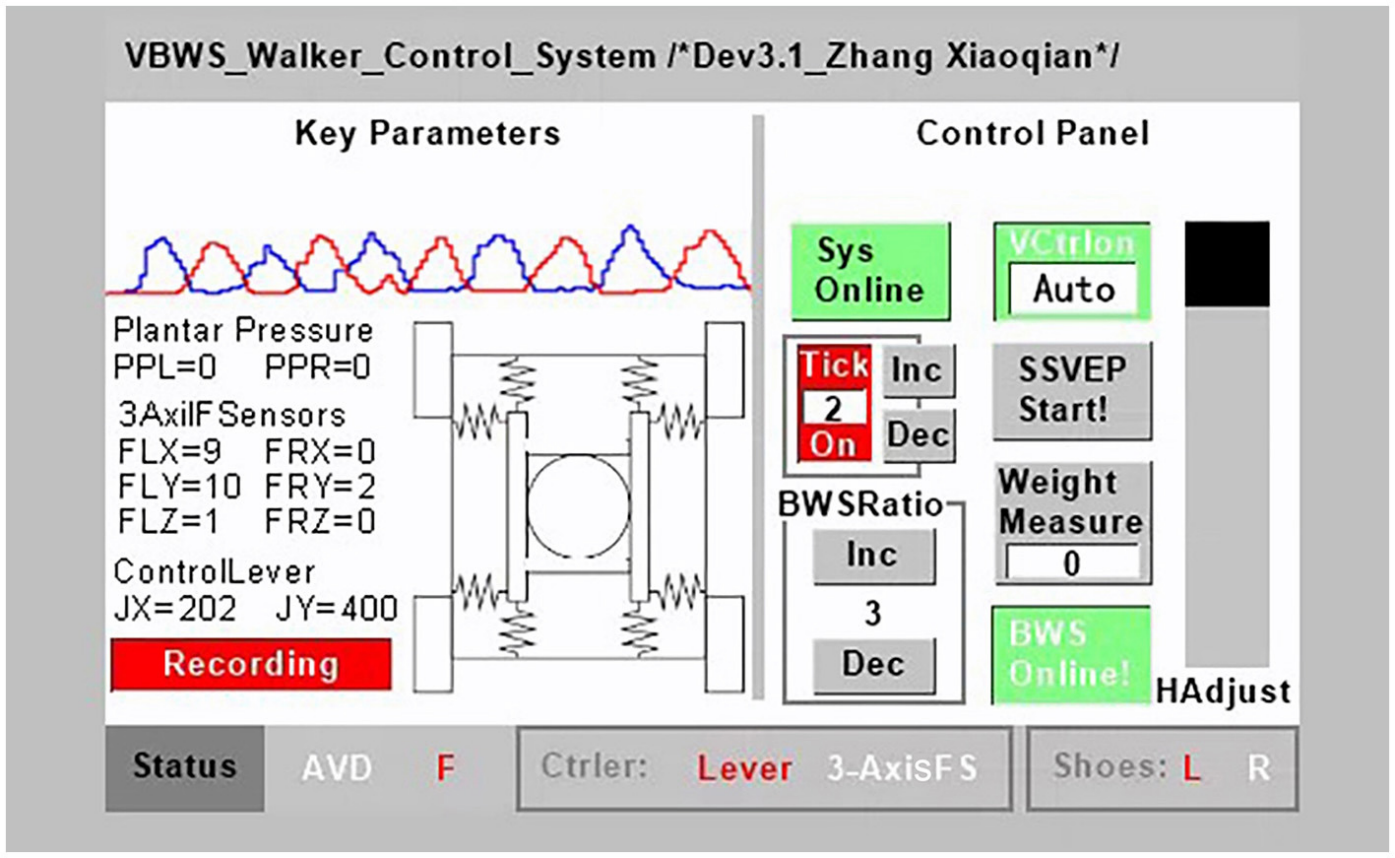
MTVBWS walker control terminal.

The shoe-based detecting system incorporates a 4-point plantar pressure sensor, model HX711, manufactured by SparkFun Electronics (USA), and a data calculation and uploads module, model LLCC68 in LoRa mode, produced by SEMTECH Corporation (USA). The HX711 pressure sensor provides a pressure acquisition resolution of 24 bits at 100 Hz, a temperature drift coefficient of ±6 nV/°C, and an output settling time of 50 ms. The LLCC68 module supports data rates for LoRaWAN ranging from SF7 to SF9 at 125 kHz, SF7 to SF10 at 250 kHz, and SF7 to SF11 at 500 kHz, which are sufficient for handling the data transmission requirements of the pressure sensor. Both the HX711 pressure sensor and the LLCC68 data calculation and upload module have been integrated into the shoe design to accurately measure the pressure distribution and provide valuable insight into the patient's gait and BWS capabilities. As shown in Figure 3, the variable names for these measurements are PPL and PPR, where PPL represents Plantar Pressure Left and PPR represents Plantar Pressure Right. These two sets of variables are input into the main control system, serving the following functions: measuring body weight before wearing the harness at the beginning of the training session, identifying gait phases during training, and consequently implementing the VUR-VBWS based on gait phase recognition. This means that during the swing phase, a doubled unloading ratio is provided. It is worth mentioning that the choice of the “doubled unloading ratio” is because an excessively large ratio would lead to abrupt changes in BWS force, which would affect the user's walking training and data collection, and the impact of which cannot be explained or eliminated at present. Conversely, a ratio that is too small would result in poor VUR-VBWS performance. The system is powered by a small and lightweight rechargeable lithium battery, making it convenient for patients to wear the shoe for data collection.

In addition, we have also developed a 3D force-sensing system for detecting the movement of the body's COM in the six-axis direction. The system is integrated on both sides of the walker's harness and comprises two three-axis force transducers of model DYDW-006 and six 485-communication analog weight transmitters of model DESENTE-D500, all manufactured by DECENT (China). These sensors can measure force in both directions of pull and push. Each of the 3D force transducers consists of a Whetstone Full-bridge circuit, which directly outputs an analog signal proportional to the magnitude of the force, with a signal range of 1.0–1.5 mV/V. The DESENTE-D500 transmitter amplifies this voltage signal to a standard analog 0–5 V/4–20 mA signal in 16 bits ^@^ 80 HZ, and each axial force-sensing signal requires one transmitter for conversion. A total of six transmitters are required for signal processing and communication with the main control system. The returned variable names are FLX, FRX, FLY, FRY, FLZ, and FRZ, as shown in Figure 3. These variables correspond to the measured forces applied by the harness on the user, where F stands for Force, L and R represent left and right, and X, Y, and Z denote the three axes. These measurements are used to calculate and track the position of the body's COM. Specifically, FLX, FRX, FLY, and FRY are used to track the COM in the horizontal direction, and then as input feedback variables to control the speed of each stepper motor. This allows the walker to track the user in the horizontal omni-direction through a combination of forward, backward, left, right, and rotational movements. Meanwhile, FLZ and FRZ are used to detect the vertical force exerted on the user by the harness, i.e., the BWS force. This force is then used as a feedback variable input to control the vertical movement of the BWS module, allowing the BWS force applied to the user to track the set BWS force value in real-time, achieving the VBWS effect.

#### 2.1.2. Horizontal omnidirectional MT mobile walker system

To achieve a vertical BWS direction and minimize the dragging effect of the walker on the patient, as well as reduce the additional burden caused by pushing and pulling the walker, it is necessary for the walker to have the ability to move in the horizontal omni-direction, including forward and backward, left and right, and rotation. Compared to the walking trajectory of healthy individuals, patients with lower limb movement disorders may exhibit abnormal gait patterns such as lateral translation and a combination of lateral translation and forward movement. Therefore, the walker needs to dynamically track the position of the human COM. To achieve this, the system consists of a Mecanum wheel drive mechanism and a control system as shown in Figure 2A. The four Mecanum wheels have a diameter of 152 mm and can support a total weight of 400 kg [Zhejiang Dongyang Century Load Wheel Manufacturing Co., Ltd. (China)]. The wheels are driven by four ASPINA-S60D540A-MC010 stepper motors manufactured by Sansha Electric Manufacturing Co., Ltd. (Japan), each featuring a holding torque of 10N^*^m (at 24V, 4A-MAX current drive) and a basic stepping angle of 0.18°. The maximum speed is 60 RPM with a 1:10 gear ratio reducer. In terms of safety, even if the driver is powered off (without holding torque) at this reduction ratio, the four wheels will not be forced to rotate, i.e., unidirectional power transmission, ensuring that accidents due to free sliding are avoided. Furthermore, the limitation on maximum speed further ensures relative safety in the event of accidental loss of control. Relevant parameters for this aspect will be provided in Section 2.2.1. The drive mechanism is positioned at the four corners of the walker and possesses the capability to drive both the patient's weight and the weight of the walker. By precisely controlling the rotation speed and direction of the four omnidirectional Mecanum wheels, the aforementioned planar omnidirectional movement capabilities, including forward and backward, left and right, and rotational motions, can be achieved. The stepper motor controller model is SAMSR-MD-2504 manufactured by Sansha Electric Manufacturing Co., Ltd. (Japan), which communicates with the main control system through level logic control, including enable signal, speed control pulse signal, and direction signal. The control panel, located in front of the patient, includes an emergency stop control, mode switching, joystick, power button, and more, providing a convenient and safe interface for patient operation. In addition, the joystick serves to facilitate the direct control of the walker's movement in certain scenarios, such as allowing staff to easily maneuver the walker. The system is powered by two 24 V, 30 Ah rechargeable lithium batteries with overload and overheating protection functions. The maximum safe discharge current of the system is 20A, and the batteries can provide a relatively stable current without interference. Moreover, the MT mode is not suitable for accidental tilting situations. Although the VBWS module of this system will halt the downward movement of the harness to prevent tipping when it detects abnormal BWS values, the emergency stop button can still be manually triggered to halt the motion of all actuators. In this case, the walker framework needs to provide horizontal support to ensure that the user does not tip over, and the framework, therefore, has a certain design load capacity redundancy to give a support force and passive safety. Relevant parameters for this aspect will be provided in Section 2.2.1.

#### 2.1.3. VBWS system based on gait characteristics

Distinct from static or SBWS systems, VBWS systems can dynamically adjust the BWS ratio based on the vertical movements of the body during walking and the BWS ratio in different gait phases. Moreover, when detecting abnormal force values, the system halts the descent of the harness module to provide support and prevent falls. Simultaneously, the framework strength must be sufficient to provide adequate upward support to ensure safety. Parameters related to this aspect will be presented in Section 2.2.1. As Figure 2C shows, the BWS system consists of two parts: a fixture and a lifting mechanism. The fixture consists of a harness, a 3D force sensor system, and straps. The straps are used to fix and support the patient's legs, hips, and waist, and are completely locked with the harness. The harness has a 3D force sensor system installed on both sides. The fixture is combined with the lifting mechanism through a detachable hinge. The lifting mechanism is divided into two groups, left and right, driven by four electric cylinders of model CNF-JXM003 manufactured by TiMOTION Technology Co., Ltd. (China), with a pushing velocity of 20 mm/s and a pushing force of 140 kg. The driver for the electric cylinder is a dual-loop feedback system of model AQMD2410NS-B3 manufactured by Chengdu Akelc Technology Co., Ltd. (China), with a rated current of 7.5A. The system communicates with the main control system through logic-level signals. The left and right groups of the lifting mechanism will simultaneously perform vertical movements, driving the harness to move. Under the control of the aforementioned feedback variables PPL, PPR, FLZ, and FRZ, the VBWS modes under the FUR and VUR settings will be achieved. When in use, staff members first remove the fixed fixture system, the patient enters the walker, staff members help install the fixture system, and finally, they securely fix the patient to the BWS system for VBWS walking control.

#### 2.1.4. System control terminal and data acquisition system

To integrate the above functions and to record the experimental data collected, we proposed a control, data acquisition, and analysis system for the training walker. Figure 2B shows the main control, wireless transmission, and drive system.

Among them, the terminal system consists of a serial screen module, a voice recognition module, a wireless transceiver module, and a customized operator interface system. The master control system adopts the STM32 F407IGT6 development board manufactured by Shenzhen Embedfire Electronics Co., Ltd. (China), and the serial interactive screen module adopts TJC4832T135_011C manufactured by Shenzhen Taojingchi Electronics Co., Ltd. (China), which has a capacitive touch screen, can be programmed, and forms a bidirectional logic control with the microcontroller system. It is connected to the microcontroller at 115,200 baud rate through TTL level, which can ensure the punctuality of data transmission. The speech recognition module adopts the LD3320-based solution with internal integration of STC11 microcontroller, which is manufactured by Shenzhen Leadtone Technology Co., Ltd. (China), to realize the recognition of specific phrases through programming, and the comprehensive recognition rate of the module is tested to be more than 80%. This was useful for the accuracy of data collection during the experiments we conducted because when the user reaches out to manually operate functions on the control panel, such as starting training, recording data, or stopping training, it can cause certain body movements. These disturbances can have a significant impact on the reliability of the experimental data. By using voice control, we were able to prevent the appearance of non-correlated body movement interference with gait events, making the experimental data more consistent with our intended purpose. In addition, the wireless transceiver module uses the HC-42 nRF52832 Bluetooth transceiver module manufactured by Guangzhou HC information technology Co., Ltd. (China), which operates at a baud rate of 115,200 and therefore allows for rapid remote transmission of experimental data to the upper computer system, and wireless transmission allows for experimental data collection within 80 m during the experiment, free from the constraints of the rope. Finally, we wrote the corresponding operation interface system control program according to the system control logic and experimental function requirements, as shown in Figure 3.

The main control interface of the terminal system is divided into three sections: left, right, and bottom, corresponding to the key parameter display area, control panel, and status display, respectively. Key parameters include real-time status and readings of various sensors during the experiment, and these parameters are used for the control of the walker system and the experimental data. Below are the wireless data upload start and stop buttons, which can be used to record valid data in the remote upper computer system at appropriate times during the experiment. Each data record includes all key parameters displayed on the interface, including system parameters, gait phases, and continuous motion data for each gait phase, recorded with time as a reference, to facilitate subsequent experimentation and analysis work. Notably, by reading the PPL and PPR values relative to time, we can obtain the fluctuation of the force on each foot or leg during different gait phases in the training, thereby allowing us to compare and evaluate the SBWS, FUR-BWS, and VUR-BWS modes stability performance of BWS gait training. By reading the FLX, FRX, FLY, FRY, FLZ, and FRZ values relative to time, we can determine the dragging force from the walker on the user in horizontal omni-directions, as well as the BWS force in the vertical direction. These values play a key role in evaluating the system's MT and the support effect of the BWS system. JX and JY represent the walker system's horizontal velocity controlled by the joystick, which serves as a data reference. Gait phases F (Flat contact), H (Heel strike), and S (Swing) will be calculated based on PPL, PPR values, measured body weight values, and BWS ratios. These gait phases are used as dividing points for recording gait data, which facilitates the analysis of the walker system's MT and VBWS performance in reference to gait phases. The control panel is an interactive operating interface. From top to bottom and left to right, it includes system operation and emergency stop control, voice control on and off, metronome on and off, including metronome frequency adjustment, reserved interface for brain–computer interface control mode, BWS ratio increase and decrease adjustment, weight measurement, automatic and manual BWS system control, and BWS system lifting and display two-in-one slider. The emergency stop control touch button and the physical emergency stop button share the same functionality. Triggering any button will cause all actuators to cut power and brake to ensure safety. The status display bar, from left to right, shows obstacle avoidance status, gait events, joystick or three-dimensional force sensor control status, and the wireless connection status between the plantar pressure shoe and the system. The infrared obstacle avoidance sensor, used for obstacle recognition, can stop forward motion when detecting obstacles ahead and illuminate the “AVD” indicator until the obstacle is cleared and user control is restored.

### 2.2. Control algorithm for MTVBWS walker

#### 2.2.1. Mathematical model in passive towing SBWS mode

**Figure 4:**
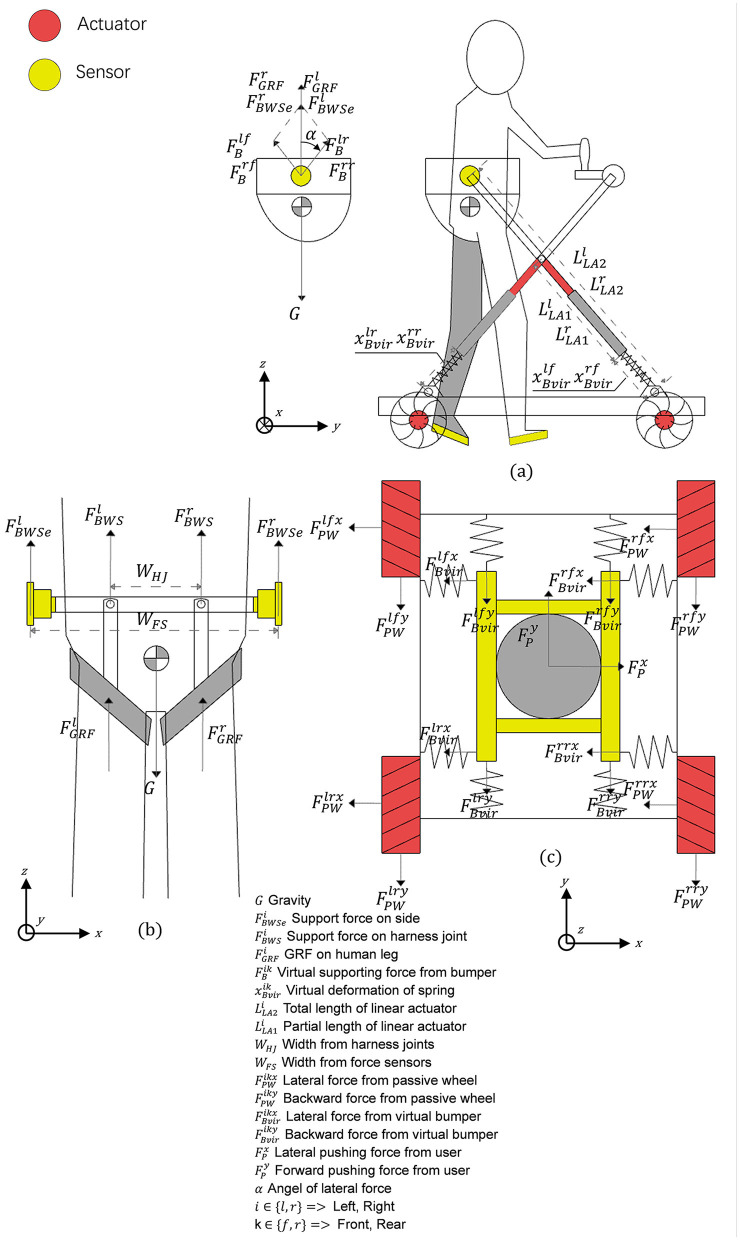
Force analysis diagram of MTVBWS walker. **(a)** Force analysis of the waist brace relative to the frame; **(b)** Force analysis of the human body relative to the waist brace; **(c)** Force analysis of the omni-direction move system.

In this study, we propose two different control modes: Passive towing SBWS mode and MTVBWS mode. When in the Passive towing SBWS mode, the system runs in a pre-set fixed BWS ratio coefficient. The walker is towed by the users from their residual strength. During the training process, the electric cylinder is maintained at a preset position, and the four Mecanum wheels rotate passively. As shown in Figure 4a, four sets of spring buffer systems articulate the base and the harness mechanisms by utilizing universal connectors. Therefore, in passive mode, the system can provide buffering for the movement of the patient's COM in 3D space during walking training, including up-and-down bouncing and horizontal swaying of the body.

First, in the vertical direction (Z-axis), since the patient is always fixed by the fixture tightly at the center of the harness of the system during training, with a small amount of sway allowed, we assume that the patient is always in the center position as shown in Figure 4b and the BWS system forms an integral part with the person, then the forces during movement can be described as:


(1) $$[F_{BWS}^{r}+F_{BWS}^{l}]+\left[F_{GRF}^{r}+F_{GRF}^{l}\right]-G=ma^{v}$$

(2) $$\left[F_{BWS}^{r}+F_{BWS}^{l}]=\text{β}G,\;\beta \in (0,1\right)$$

Where $F_{BWS}^{r}$ and $F_{BWS}^{l}$ are the final BWS force of the BWS system acting on the clamps located on both legs, $F_{GRF}^{r}$ and $F_{GRF}^{l}$ are the ground reaction force of the legs in contact with the ground, and *m* with *G* represents the patient's original weight and gravity. We define β as the VBWS scaling factor, obtained from the ratio of BWS force to body weight, and its value is determined by a pre-set minimum value in conjunction with the gait phase. *a*^*v*^ refers to the acceleration of the patient's body in the vertical direction (Z-axis). The BWS system is connected to both ends of the waist fixture through one of the two motorized actuators so that the actual BWS shown in the front view is located on the same connecting rod as the BWS acting on both ends of the fixture so that the actual BWS is:


(3) $$\begin{array}{l} F_{BWS}^{l}=F_{BWSe}^{l}\left[\frac{W_{FS}+W_{HJ}}{2w_{FS}}\right] \end{array}$$

(4) $$\begin{array}{l} F_{BWS}^{r}=F_{BWSe}^{r}\left[\frac{W_{FS}+W_{HJ}}{2w_{FS}}\right] \end{array}$$

Where $F_{BWSe}^{r}$ and $F_{BWSe}^{l}$ are the lifting force in the vertical direction (Z-axis) of the electric cylinders acting on one end of the waist fixture, and *W*_*HJ*_ is the distance between the two fixed points of the fixture and the harness, and *w*_*FS*_ is the distance between the two articulation points of the electric cylinder and the fixture. The forces $F_{BWSe}^{r}$ and $F_{BWSe}^{l}$ are provided by the two electric cylinders, arranged in a crossed configuration and hinged together at a distance $L_{LA1}^{r}$ from the fixed end. Each set of electric cylinders moves simultaneously, so that regardless of their lift, as shown in Figure 4a, the left and right split forces provided by them are the same as the vertical lifting force ($F_{BWSe}^{r}$ with $F_{BWSe}^{l}$) at an equal angle. The lifting force can therefore be described as:


(5) $$\begin{array}{l} F_{BWSe}^{l}=\frac{F_{B}^{lf}+F_{B}^{lr}}{\cos\alpha } \end{array}$$

(6) $$\begin{array}{l} F_{BWSe}^{r}=\frac{F_{B}^{rf}+F_{B}^{rr}}{\cos\alpha } \end{array}$$

Where $F_{B}^{lf},\;F_{B}^{lr}$ and $F_{B}^{rf},\;F_{B}^{rr}$ are the lifting fractions in the direction of the left and right sets of crossed electric cylinders, respectively. The $F_{B}^{lf}$ with $F_{B}^{rf}$ is directly provided by the buffer, while due to the presence of the articulation, $F_{B}^{lr}$ and $F_{B}^{rr}$ need to be obtained by conversion, which is solved as follows:


(7) $$\begin{array}{l} F_{B}^{lf}=F_{S}^{lf}=-k^{f}x_{Bvir}^{lf} \end{array}$$

(8) $$\begin{array}{l} F_{B}^{rf}=F_{S}^{rf}=-k^{f}x_{Bvir}^{rf} \end{array}$$

(9) $$\begin{array}{l} F_{B}^{lr}=F_{S}^{lr}\left[\frac{L_{LA1}^{l}+x_{Bvir}^{lr}}{L_{LA2}^{l}}\right] \end{array}$$

(10) $$\begin{array}{l} F_{S}^{lr}=-k^{r}x_{Bvir}^{lr} \end{array}$$

(11) $$\begin{array}{l} F_{B}^{rr}=F_{S}^{rr}\left[\frac{L_{LA1}^{r}+x_{Bvir}^{rr}}{L_{LA2}^{r}}\right] \end{array}$$

(12) $$\begin{array}{l} F_{S}^{rr}=-k^{r}x_{Bvir}^{rr} \end{array}$$

Where ${F_{S}^{lf},F}_{S}^{rf},{F_{S}^{lr},F}_{S}^{rr}$ are the equivalent forces located in the left front, right front, left rear, and right rear buffers of the system, respectively, and $x_{Bvir}^{lf},x_{Bvir}^{rf},x_{Bvir}^{lr},x_{Bvir}^{rr}$ are their equivalent form of variables, which are negative when they are compressed, respectively, *k*^*f*^ is its stiffness coefficient located in the front two groups, and *k*^*r*^ is the stiffness coefficient of the two groups located at the rear. To keep the front and rear balance when the harness is under downward pressure, i.e., the front and rear drop heights are the same, different stiffness coefficients are needed for the front and rear buffers, which is as follows:


(13) $$\begin{array}{l} F_{B}^{lf}=F_{S}^{lf}={F_{B}^{lr}=F}_{S}^{lr}\left[\frac{L_{LA1}^{l}+x_{Bvir}^{lr}}{L_{LA2}^{l}}\right] \end{array}$$

Second, in the plane direction (X-axis and Y-axis), as shown in Figure 4c, we assume that the patient and the BWS system move simultaneously in this plane direction relative to the walker chassis and the ground, and the BWS system is connected to the walker chassis by buffers, which we equate here to a total of 8 sets of spring cushion structures located in the front and rear. The forces during its movement can be described as follows:


(14) $$\begin{array}{l} F_{P}^{x}=F_{Bvir}^{ikx}+F_{PW}^{ikx},\;i\in \left\{l,r\right\},\;k\in \left\{f,r\right\} \end{array}$$

(15) $$\begin{array}{l} F_{Bvir}^{ikx}=-k_{1}x_{Bvir}^{ikx},\;i\in \left\{l,r\right\},\;k\in \left\{f,r\right\} \end{array}$$

(16) $$\begin{array}{l} F_{P}^{y}=F_{Bvir}^{iky}+F_{PW}^{iky},\;i\in \left\{l,r\right\},\;k\in \left\{f,r\right\} \end{array}$$

(17) $$\begin{array}{l} F_{Bvir}^{iky}=-k_{2}x_{Bvir}^{iky},\;i\in \left\{l,r\right\},\;k\in \left\{f,r\right\} \end{array}$$

Where $F_{P}^{x},F_{P}^{y}$ are the lateral (X-axis) and anteroposterior (Y-axis) forces of the patient relative to the BWS system, $F_{Bvir}^{ikx},F_{Bvir}^{iky}$ are the lateral and anterior-posterior reaction force of the cushioning system, $F_{PW}^{ikx},F_{PW}^{iky}$ are the lateral and anterior-posterior reaction force of the tire relative to the system during passive rotation, *k*_1_, *k*_2_ are the lateral and anteroposterior equivalent stiffness coefficient of the cushion, and $x_{Bvir}^{ikx},x_{Bvir}^{iky}$ is the lateral and anterior-posterior equivalent deformation variable of the buffer, which is negative when it is compressed. Table 1 shows the key features of the developed MTVBWS walker system. The maximum designed load-bearing capacity of the device structure represents the maximum force that the walker frame can withstand in all directions. To ensure user safety, the design has a certain degree of redundancy. The maximum 3D directional movement speed of the device represents the ultimate speed achieved by the selected actuators, including the four Mecanum wheels along the X and Y axes after the reducer, and the maximum combined speed on the Z-axis achieved by the four linear worm gear actuators. Similarly, considering user safety, even in the case of uncontrolled accidental motion, such maximum speeds will allow the user or an assistant enough reaction time to intervene, for example, by triggering an emergency stop.

**Table 1:** Key features of the developed MTVBWS walker system.

**Features**	**Parameters**
MTVBWS walker total weight (*m*_*Total*_)	105 [Kg]
MTVBWS walker total maximum bearing	Approximately 400 [Kg]
MTVBWS walker external height	900–1,500 [mm]
MTVBWS walker external width	850 [mm]
MTVBWS walker inner width	630 [mm]
MTVBWS walker external length	1,100 [mm]
MTVBWS module weight (*m*_*BWS*_)	25 [Kg]
Maximum BWS force	Approximately 1,300 [N] ^@^ Z-axis
Maximum planar driving force	Approximately 500 [N] ^@^ X-axis and Y-axis
Frame maximum design load capacity	1,500 [N] ^@^ X-axis and Y-axis, 2,500 [N] ^@^ Z-axis
Maximum 3D movement speed (maximum speed of actuators)	1,000 [mm/s] ^@^ X-axis and Y-axis, 230 [mm/s] ^@^ Z-axis

#### 2.2.2. Control methods in VBWS mode

In the MTVBWS mode, the BWS system changes the BWS preload ratio by actively moving the fixture in the vertical direction during the training process. This system will partially compensate for the instability of the BWS ratio caused by the delayed action response of the electric actuator due to the buffer described previously.

**Figure 5:**
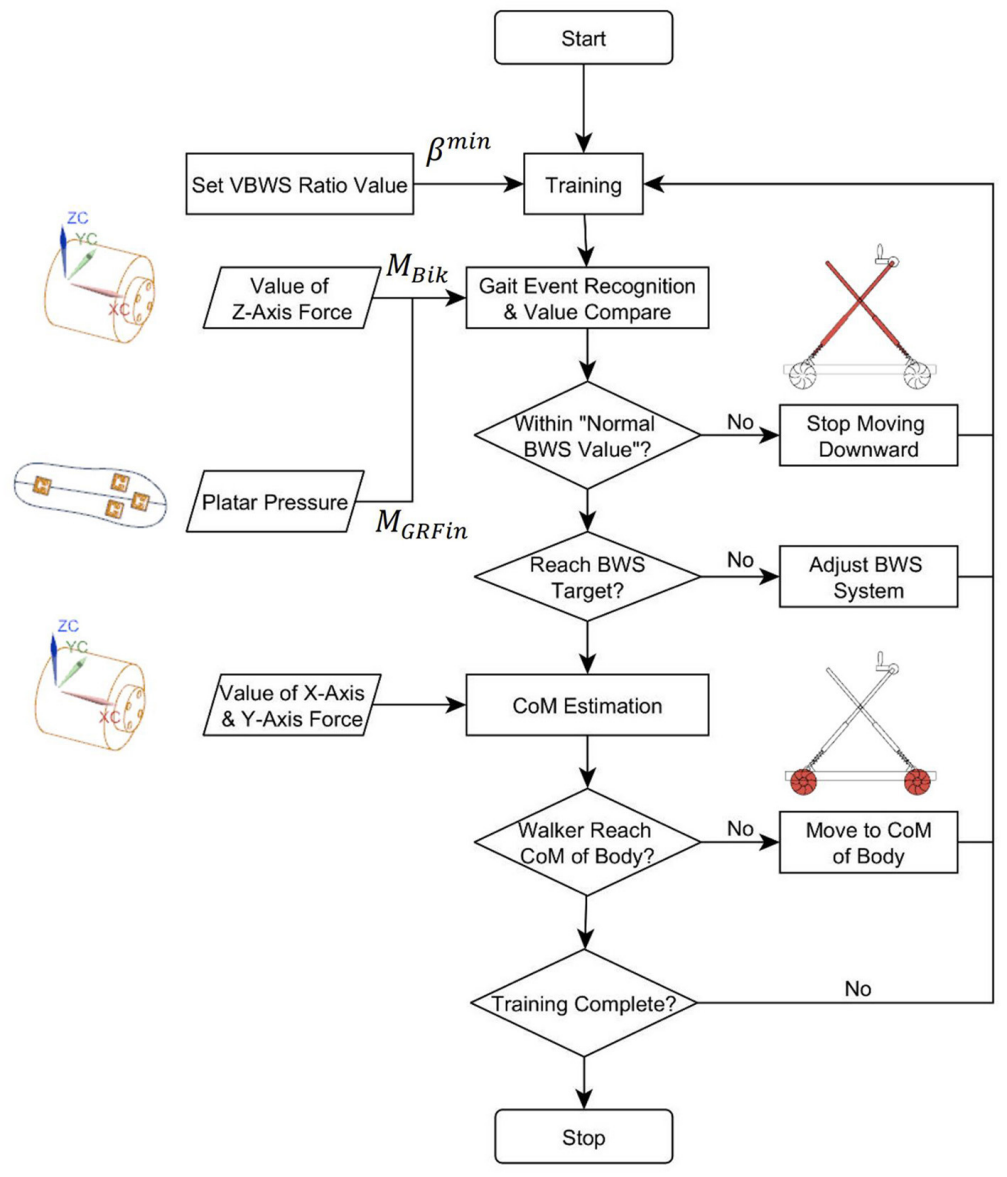
Control flow of MTVBWS walker.

The system control logic is shown in Figure 5, and the minimum BWS ratio β^min^ needs to be set before training, and the β^min^ stands for the minimum BWS ratio in the Stance Phase, which can be described as follows:


(18) $$\begin{array}{l} \beta^{\min}=\frac{F_{BWS}^{\min}}{G} \end{array}$$

Where $F_{BWS}^{\min}$ is the minimum BWS target value under the stance phase. To start the training, the system will continuously read the data from two 3D force sensors and the plantar pressure sensors, of which the vertical force *M*_*GRFi*_ will be used in the BWS system. During the real-time operation, we collect and track the last 5 sets of data sent by each sensor separately into a datasheet, which can be described as follows:


(19) $$\begin{array}{lll} M_{Bik} & \in & \left\{M_{Bik}^{t},M_{Bik}^{t-1},M_{Bik}^{t-2},M_{Bik}^{t-3},M_{Bik}^{t-4}\right\},\;i\in \left\{l,r\right\},\;k\in \left\{f,r\right\} \end{array}$$

(20) $$\begin{array}{l} M_{Bik}^{Avgt}=\frac{M_{Bik}^{t}+M_{Bik}^{t-1}+M_{Bik}^{t-2}+M_{Bik}^{t-3}+M_{Bik}^{t-4}}{5} \end{array}$$

(21) $$\begin{array}{lll} M_{GRFi} & \in & \left\{M_{GRFi}^{t}{,M}_{GRFi}^{t-1},M_{GRFi}^{t-2},M_{GRFi}^{t-3},M_{GRFi}^{t-4}\right\},i\in \left\{l,r\right\} \end{array}$$

(22) $$\begin{array}{l} M_{GRFi}^{Avgt}=\frac{M_{GRFi}^{t}{+M}_{GRFi}^{t-1}+M_{GRFi}^{t-2}+M_{GRFi}^{t-3}+M_{GRFi}^{t-4}}{5} \end{array}$$

By analyzing this data, we can obtain the average of the last 5 sets of pressure values of the 3D force sensor in the vertical direction $M_{Bik}^{Avgt}$ and the average of the last five sets of pressure values of the plantar pressure sensor in each foot $M_{GRFi}^{Avgt}$. The values are recorded every 5 ms for conversion. If the absolute value of $M_{Bik}^{Avgt}$ is greater than the measured relative user weight value, indicating an “abnormal BWS value,” the system considers that the user tends to fall. This process can be expressed as:


(23) $$\begin{array}{l} M_{Bik}^{Avgt}>\frac{2}{3}G \end{array}$$

At this point, the VBWS system immediately ceases its downward motion, providing support to the user to prevent falls. Beyond this, the system operates under normal VBWS mode, i.e., within the “normal BWS value.” By further processing the data, we can determine the foot-ground contact characteristics. The calculation can be described as:


(24) $$\begin{array}{l} \text{Lift Up}.\;\frac{dM_{GRFi}^{Avgt}}{dt}<0\;\&\&\frac{dM_{GRFi}^{Avgt-1}}{dt-1}<0 \end{array}$$

(25) $$\begin{array}{l} \text{Support for Steady Contact}.\;\frac{dM_{GRFi}^{Avgt}}{dt}=0\;\&\&\frac{dM_{GRFi}^{Avgt-1}}{dt-1}=0 \end{array}$$

(26) $$\begin{array}{l} \text{Landing Press Down}.\;\frac{dM_{GRFi}^{Avgt}}{dt}>0\;\&\&\frac{dM_{GRFi}^{Avgt-1}}{dt-1}>0 \end{array}$$

Based on the trends of plantar pressure values over time, we can classify them into four trends of foot lift, support, and foot drop, and combined with the average values of plantar pressure, we can further define each gait phase. Here, we apply the gait classification method proposed by V. Agostini et al. to divide a gait cycle into four subphases, defined as heel contact phase (H), flat foot contact phase (F), push-off or heel-off phase (P) and swing phase (S), which are as follows:


(27) $$\begin{array}{l} \text{S}\to H:Press\;Down\;\&\&{\;\frac{G}{6}\geq M}_{GRFi}^{Avgt}{\;\&\&\;M}_{GRFi}^{Avgt}>\frac{G}{30} \end{array}$$

(28) $$\begin{array}{l} \beta^{set}={2\beta }^{\min} \end{array}$$

(29) $$\begin{array}{l} \text{H}\to F:Press\;Down\;\&\&\;\frac{G}{4}\geq M_{GRFi}^{Avgt}\;\&\&{\;M}_{GRFi}^{Avgt}>\frac{G}{6} \end{array}$$

(30) $$\begin{array}{l} \beta^{set}={1.5\beta }^{\min} \end{array}$$

(31) $$\begin{array}{l} \text{F}:Steady\;Contact\&\&M_{GRFi}^{Avgt}>\frac{G}{4} \end{array}$$

(32) $$\begin{array}{l} \beta^{set}=\beta^{\min} \end{array}$$

(33) $$\begin{array}{l} \text{F}\to P:Lift\;Up\;\&\&{\;\frac{G}{4}\geq M}_{GRFi}^{Avgt}{\;\&\&\;M}_{GRFi}^{Avgt}>\frac{G}{6} \end{array}$$

(34) $$\begin{array}{l} \beta^{set}={1.5\beta }^{\min} \end{array}$$

(35) $$\begin{array}{l} \text{P}\to S:Press\;Down\;\&\&{\;\frac{G}{6}\geq M}_{GRFi}^{Avgt}{\;\&\&\;M}_{GRFi}^{Avgt}>\frac{G}{30} \end{array}$$

(36) $$\begin{array}{l} \beta^{set}={2\beta }^{\min} \end{array}$$

(37) $$\begin{array}{l} \text{S}:M_{GRFi}^{Avgt}<\frac{G}{30} \end{array}$$

(38) $$\begin{array}{l} \beta^{set}=2\beta^{\min} \end{array}$$

**Figure 6:**
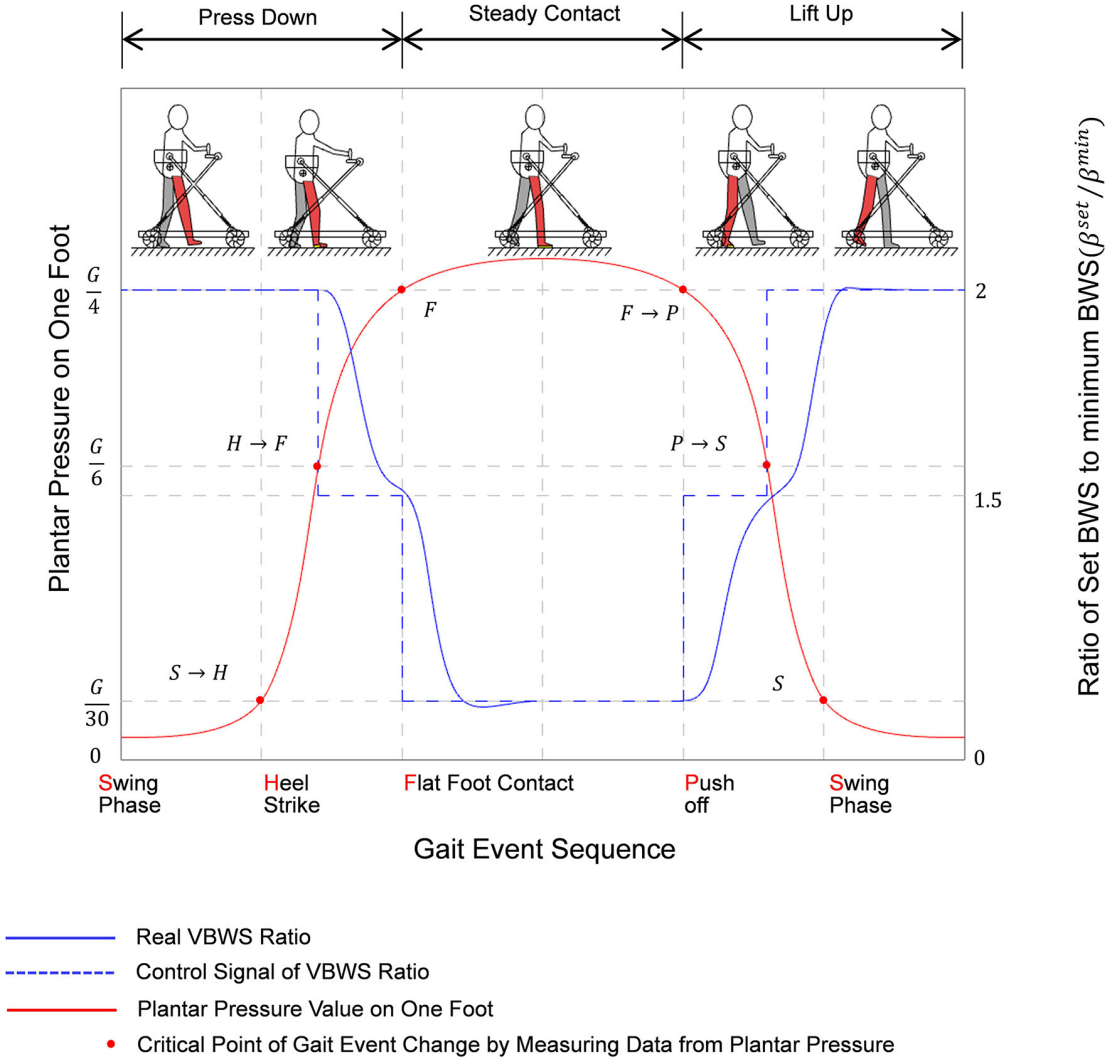
Variation diagram of plantar pressure and ratio of set BWS corresponding to gait event sequence of a step while training with MTVBWS walker.

We defined the gait event transitions and states for a given lower limb, divided into four state transition phases and two continuous state phases, and judged by the upward and downward trend of plantar pressure values in ratio to body weight values. At the same time, the control system sets up to apply at each stage a percentage of BWS β^min^ relative to the initial set value of BWS β^*set*^ at different multipliers. The gait phase recognition classification and VBWS logic are shown in Figure 6.

Noteworthy is that the actual BWS ratio does not change abruptly according to the control signal because of the spring buffer force cushioning effect and the presence of electric actuator response time, and therefore the system is more linear in response to different BWS ratios, effectively reducing the stutter in the adjustment process, and the patient, therefore, feels less abrupt changes in BWS strength. The actual BWS ratio characteristics can be described by the gait phase as follows:


(39) $$\begin{array}{l} a^{v}=a_{L}+a_{B} \end{array}$$

(40) $$\left[F_{BWS}^{r}+F_{BWS}^{l}]=\text{β}G\;\beta \in (0,1\right)$$

(41) $$\begin{array}{l} \left[F_{BWS}^{r}+F_{BWS}^{l}\right]+\left[F_{GRF}^{r}+F_{GRF}^{l}\right]+\frac{\Delta \beta }{G}-G=ma^{v} \end{array}$$

(42) $$\begin{array}{l} \text{H}\to F/F:\Delta \beta^{set}=-0.5\beta^{\min} \end{array}$$

(43) $$\begin{array}{l} \text{F}\to P/P\to S:\Delta \beta^{set}=0.5\beta^{\min} \end{array}$$

Where the acceleration of the patient's body is in the vertical direction *a*^*v*^ under the joint action of the spring buffer and the electric actuator, it is the acceleration of both *a*_*B*_, *a*_*L*_, i.e., the sum of the two. And the change values of the BWS ratio in the phase H → F/FΔβ^*set*^ are −0.5β^min^. The values of the change in the phase F → P/P → SΔβ^*set*^ are 0.5β^min^. Therefore, we can see that the overall acceleration of the BWS system *a*^*v*^ and the actual BWS ratio change value β will be compensated by the acceleration of the spring buffer and the electric actuator together.

#### 2.2.3. Control methods in MT mode

**Figure 7:**
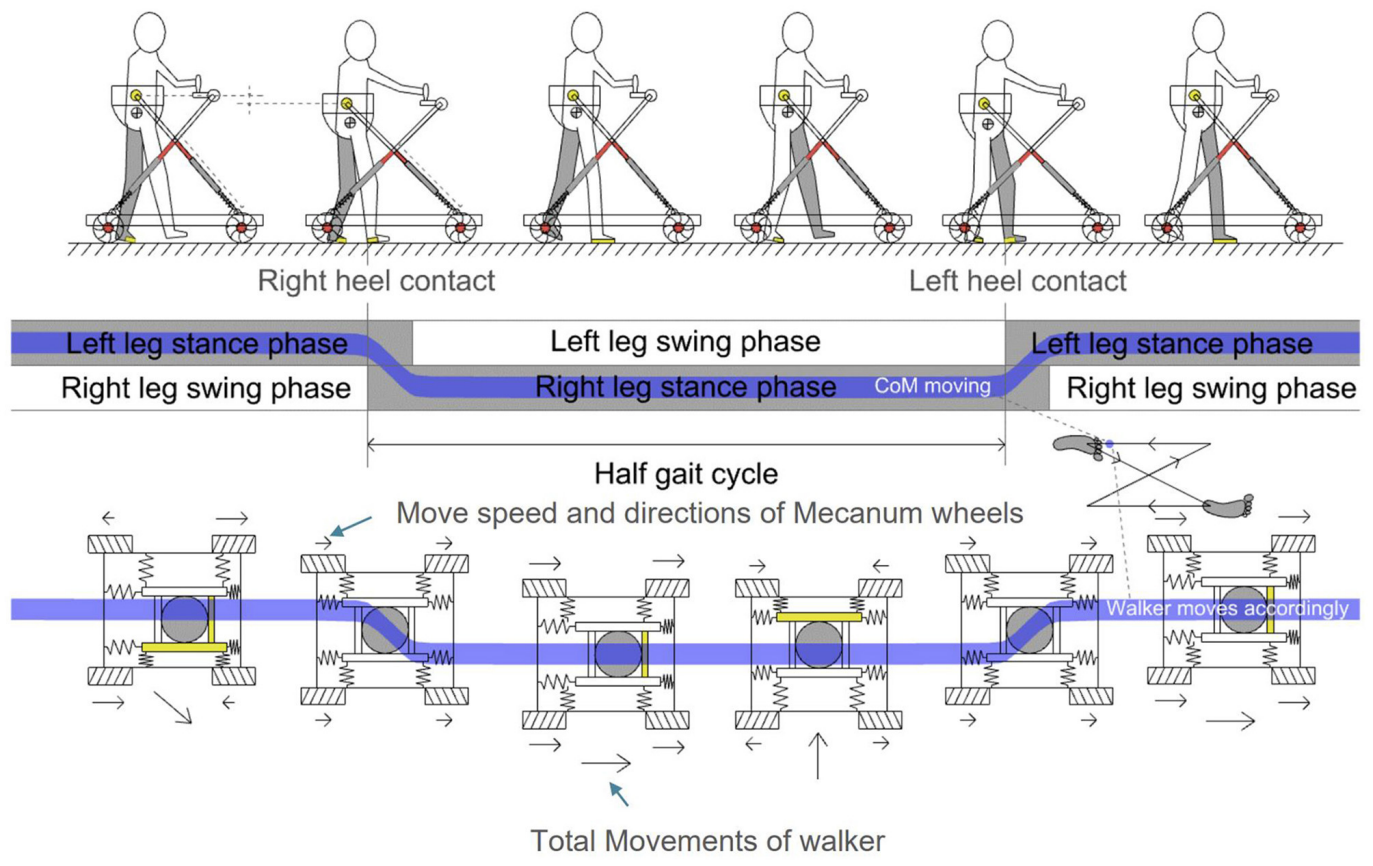
Omni-direction movements of MTVBWS walker in MT mode, corresponding to body's COM of a step event.

As shown in Figure 7, during lower limb walking training in MTVBWS mode, the walker will not only perform VBWS in the vertical direction but will also actively follow the patient's COM in the planar direction, owing to the power-driven Macanum wheels and control algorithms. Similarly, the system will partially compensate for the delayed movement response of the MT motion due to the buffer effect described above.

As shown in Figure 5, to start the training process, the system will record and analyze the force received by the 3D force sensor in the plane direction, i.e., along the X-axis and Y-axis directions. The position of the patient's COM in the plane direction is also estimated for tracking. Similar to the analysis method of the BWS system in Section 2.2.2, during the real-time operation, we collect, track, and record the forces on X-axis and Y-axis for these two 3D force sensors, respectively, and average the last 5 sets of data. The calculation can be described as follows:


(44) $$\begin{array}{lll} M_{Pik} & \in & \left\{M_{Pik}^{t},M_{Pik}^{t-1},M_{Pik}^{t-2},M_{Pik}^{t-3},M_{Pik}^{t-4}\right\},\;i\in \left\{l,r\right\},\;k\in \left\{x,y\right\} \end{array}$$

(45) $$\begin{array}{l} M_{Pik}^{Avgt}=\frac{M_{Pik}^{t}+M_{Pik}^{t-1}+M_{Pik}^{t-2}+M_{Pik}^{t-3}+M_{Pik}^{t-4}}{5} \end{array}$$

(46) $$\begin{array}{l} F_{P}^{x}=M_{Plx}+M_{Prx} \end{array}$$

(47) $$\begin{array}{l} F_{P}^{y}=M_{Ply}+M_{Pry} \end{array}$$

**Figure 8:**
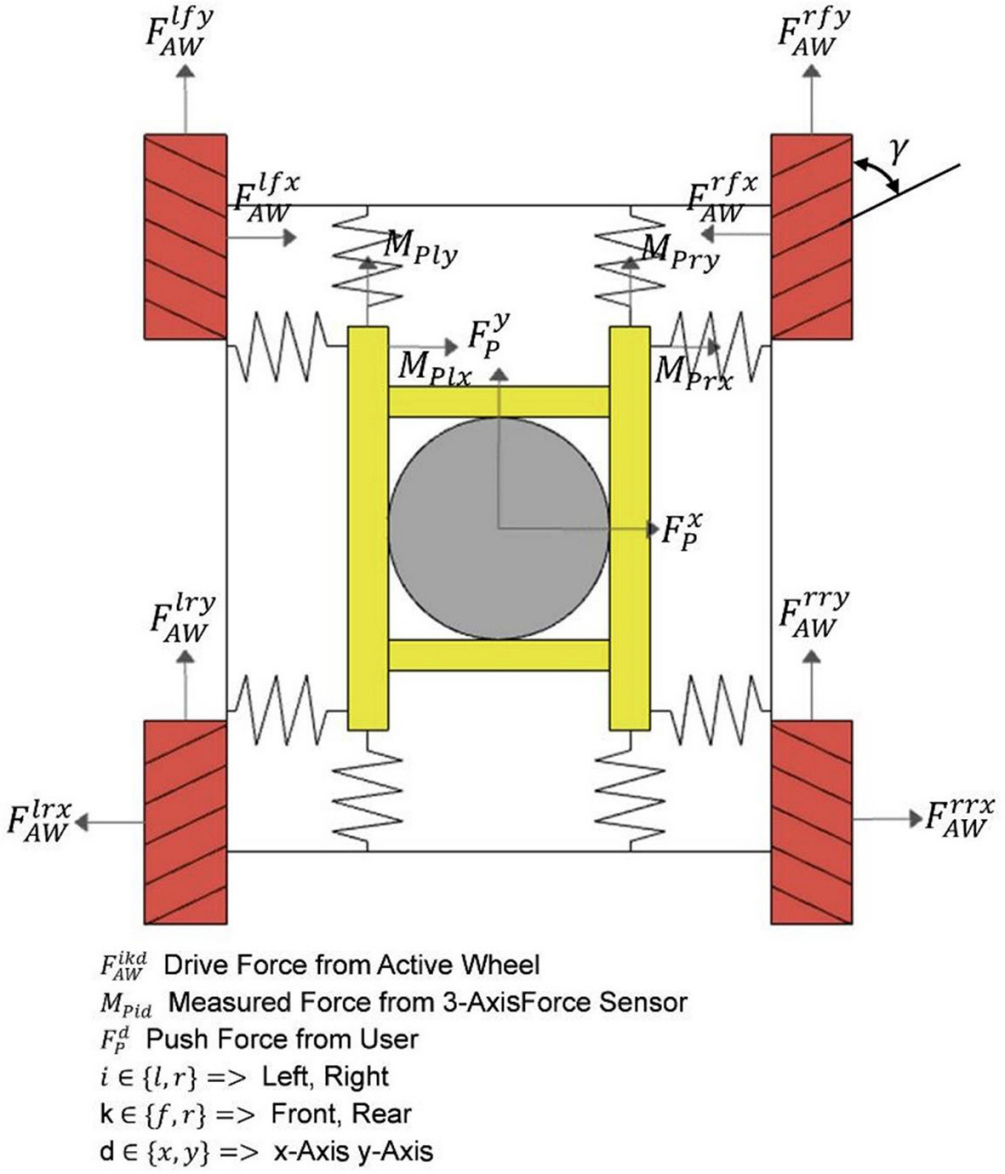
Detailed force analysis of omni-direction move system.

Where $M_{Pik}^{Avgt}$ is the average of the last five sets of force values, *i*∈{*l, r*} refers to the left and right 3D force sensors, and *k*∈{*x, y*} refers to the measurement of the force along the X-axis or Y-axis direction, and the value is recorded every 5 ms for conversion. This value is generated by the human action on the walker, i.e., $F_{P}^{x},\;F_{P}^{y}$ are the final expected walker drive forces, i.e., the combined force generated by all Macanum wheels, as shown in Figure 8.

And depending on the magnitude, direction, and point of action of the input sensor force, the leftward and rightward excursions and rotations in the relative forward direction can be achieved by configuring the four wheels with different driving forces, and the relationship can be expressed as follows:


(48) $$\begin{array}{l} F_{P}^{x}=M_{Pld}+M_{Prd},d\;\in \left\{x,y\right\} \end{array}$$

(49) $$\begin{array}{l} M_{Pld}^{Avgt}+M_{Prd}^{Avgt}=F_{AW}^{lfd}+F_{AW}^{rfd}+F_{AW}^{lrd}+F_{AW}^{rrd},d\\ \in \left\{x,y\right\} \end{array}$$

(50) $$\begin{array}{l} F_{AW}^{ikx}=F_{AW}^{iky}\tan\gamma ,i\in \left\{l,r\right\},\;k\;\in \left\{f,r\right\} \end{array}$$

(51) $$\begin{array}{l} \text{Shift Left}:M_{Plx}+M_{Prx}<0\;=>\;{|F}_{AW}^{lfx}+F_{AW}^{rrx}|<{|F}_{AW}^{rfx}\\ +F_{AW}^{lrx}| \end{array}$$

(52) $$\begin{array}{l} \text{Shift Right}:M_{Plx}+M_{Prx}>0\;=>\;{|F}_{AW}^{lfx}+F_{AW}^{rrx}|>{|F}_{AW}^{rfx}\\ +F_{AW}^{lrx}| \end{array}$$

(53) $$\begin{array}{l} \text{Turn Left}:M_{Ply}<M_{Pry}\;=>\;{|F}_{AW}^{lfy}+F_{AW}^{lry}|<{|F}_{AW}^{rfy}+F_{AW}^{rry}| \end{array}$$

(54) $$\begin{array}{l} \text{Turn Right}:M_{Ply}>M_{Pry}\;=>\;{|F}_{AW}^{lfy}+F_{AW}^{lry}|>{|F}_{AW}^{rfy}+F_{AW}^{rry}| \end{array}$$

**Figure 9:**
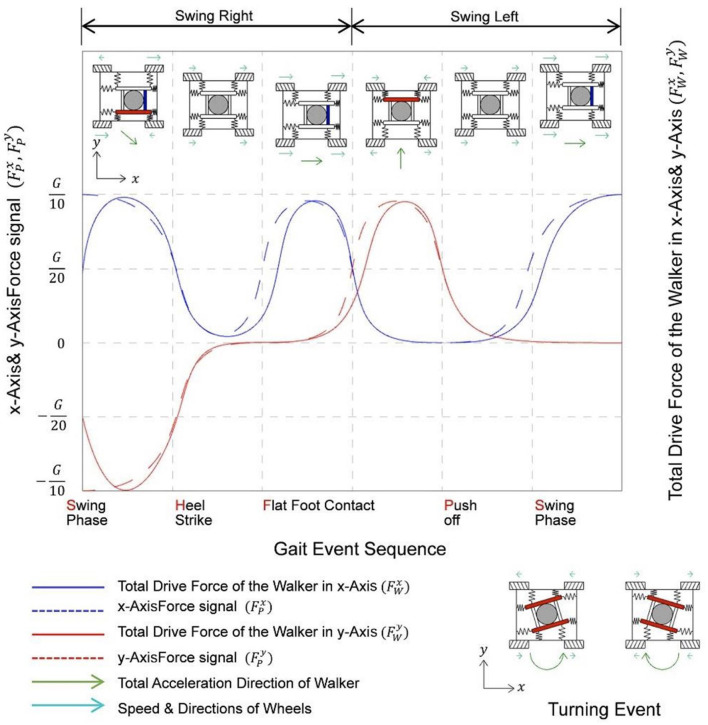
Variation diagram of force signal in X, Y axes, and corresponding drive force of a step while training with MTVBWS walker.

Where $F_{AW}^{ikx},\;F_{AW}^{iky}$ are the driving forces configured on each wheel. Again, the four subphases of a gait cycle described previously were used as the basis for analysis of the general characteristics of the motion of the walker during training in the horizontal direction, following the patient's body motion during a gait cycle, as shown in Figure 9.

It can be seen that during a gait cycle, as the patient walks forward, the right and left feet alternately enter the swing and stance states, and the body's COM will swing from side to side. If we define the starting point to be the swing state of the right foot, the patient's COM will swing to the right during the period from the swing state of the right foot to the flat foot contact of the right foot. The patient's COM will swing to the left during the period between the push-off of the right foot and the switch to full foot contact with the left foot, during which the patient's COM will swing to the left. By capturing the movement of this COM, the walker is controlled to swing left and right synchronously. It is important to note that the actual swing of the walker will be slightly delayed from the patient's body swing, and the drag force from the delay will be partially offset by the equivalent spring cushion. Assuming that the driving force loaded on the wheel is set equal to the actual driving force, we know the force characteristics of the system when following, which can be described as follows:


(55) $$\begin{array}{l} F_{P}^{d}=[F_{AW}^{lfd}+F_{AW}^{rfd}+F_{AW}^{lrd}+F_{AW}^{rrd}]+[F_{Bvir}^{lfd}+F_{Bvir}^{rfd}\\ \;+F_{Bvir}^{lrd}+F_{Bvir}^{rrd}],d\;\in \{x,y\} \end{array}$$

During the training process, turning events also occur. By collecting the filtered force values of two sets of 3D force sensors on the X-axis, denoted as $M_{Ply}^{Avgt},M_{Pry}^{Avgt}$, the difference between these values is calculated to determine the amount of rotation. This information is then used to control the motion of the four wheels at different speeds and directions, causing the walker to rotate, as shown in Figure 9. Since this event is not within the standard gait event we defined, it will not be discussed further.

## 3. Experiment results and discussion

### 3.1. Conduction of the experiments

We experimentally validated the user's ground walking performance with the assistance of this walker under various modes, focusing on evaluating its effectiveness in providing walking support and examining the safety aspect of the walker system during the training process. Our evaluation criteria encompassed the VUR-VBWS effect, MT effect, and safety assurance. For the VUR-VBWS effect validation, pressure shoe data were collected to obtain the time-varying PPL and PPR values as well as gait phase changes, comparing the differences in different gait phases under SBWS, FUR-VBWS, and VUR-VBWS modes. This aimed to demonstrate the superiority of the VUR-VBWS mode over other modes in reducing fluctuations in leg BWS forces. For the MT effect validation, 3D force sensors were used to obtain FLX, FRX, FLY, and FRY data, which represent the time-varying horizontal resistive forces experienced by the user during the walking training process with the walker. The comparison of these values over time in MT mode (active tracking) and Passive towing mode (non-active tracking) was used to verify that the MT mode is more effective in reducing resistive forces. For safety assurance validation, we assessed the system's performance under extreme conditions by testing various aspects. First, by using the rotational speed data returned from the four stepper motor encoders, we calculated the combined velocity in the horizontal omni-direction, which represents the walker's speed, and determined its maximum achievable speed. This was used to evaluate system safety in one dimension. Second, the sliding distance after power-off was directly obtained through on-site distance measurement, which assessed the safety performance in the emergency stop performance dimension. The experimental objectives, methods, and data processing were fully explained to the subjects, and written consent was approved by the subjects to sign the experiment agreements. The study was approved by the Institutional Review Board (protocol code SIAT-IRB-221215-H0634). Five able-bodied subjects were selected for this experiment (gender: male; age at the time ± standard deviation, 22.6 ± 1.2; height, 167.2 ± 8.3 cm; and weight, 62.6 ± 5.2 kg). At the same time, we calibrated the sensors that had an impact on the experimental data before each experiment. The DESENTE-D500 transducer used for measuring 3D force was calibrated using a combination of weights, while the HX711 excitation module used for measuring plantar pressure was set to automatically calibrate. It automatically calibrated each time it was powered on before the experiment to ensure the reliability of the experimental data.

Before the start of each experiment, subjects were required to wear special plantar pressure detection shoes and BWS harnesses and confirm the correctness of wearing to ensure data accuracy. In the functional training validation experiments, the experiment was divided into SBWS walking, FUR-VBWS walking, VUR-VBWS walking, MT mode walking, and Passive towing mode walking. In the SBWS, FUR-VBWS, and VUR-VBWS modes, the MT mode will be used to include data from the MTVBWS mode, with the BWS ratio set to 20% and 30%. Similarly, in the MT and Passive towing modes, the VUR-VBWS mode is used with a 20% BWS ratio. Each user was asked to take 15 steps in a normal walking posture on a flat indoor road at a fixed pace for each experiment session. Safety experiments included maximum movement speed tests and instantaneous stopping sliding distance tests. It is worth noting that the safety performance of the active obstacle avoidance function, which is implemented similarly to the emergency stop function by cutting off power to the actuator, can refer to experimental data under emergency stop conditions. In the safety experiments, five subjects were asked to walk forward (along the Y-axis) at their maximum speed in MT mode for 15 m and to walk laterally (along the X-axis) at their maximum speed for 1 m to the left and right. Each subject experimented five times, and the maximum speed and corresponding forces during the five walking sessions were calculated using the velocity feedback from the motor speed encoder and the force feedback from the 3D force sensor. Simultaneously, at the endpoint of the walk, the subjects were instructed to press the emergency stop button, recording the instantaneous velocity and force at the time of pressing, as well as the distance moved from pressing to complete stopping, to verify the effectiveness of the emergency stop. Additionally, the five subjects were asked to perform instantaneous squatting actions under the VBWS (with both FUR and VUR settings) mode, with the experiment conducted five times. The protective efficacy against accidental falls was validated by measuring the downward displacement of the harness module from the moment the subject began to relax until the harness came to a complete stop.

### 3.2. Result and discussion

We collected data from all the sensors through the embedded system and sent this data to the upper PC at 20 Hz through wireless serial communication, which recorded all the data of the experiment. We chose a data acquisition frequency of 20 Hz, as it represented a balance between achieving a rich data set within a short time frame and ensuring system stability. We found that higher frequencies led to instability and potential data loss in our system due to the large volume of data being transmitted. Thus, 20 Hz was determined to be the highest feasible frequency for our setup, providing sufficient data for analysis while maintaining system reliability, as supported by previous research (Giggins et al., 2014). With this data, we calculated the support force provided by each foot of the subject, i.e., plantar pressure, BWS force provided by the 3D sensors in the Z-axis, and lateral force provided by the 3D sensors in the XY-axis, and recorded them against time as well as gait events.

#### 3.2.1. Feasibility of VBWS

**Figure 10:**
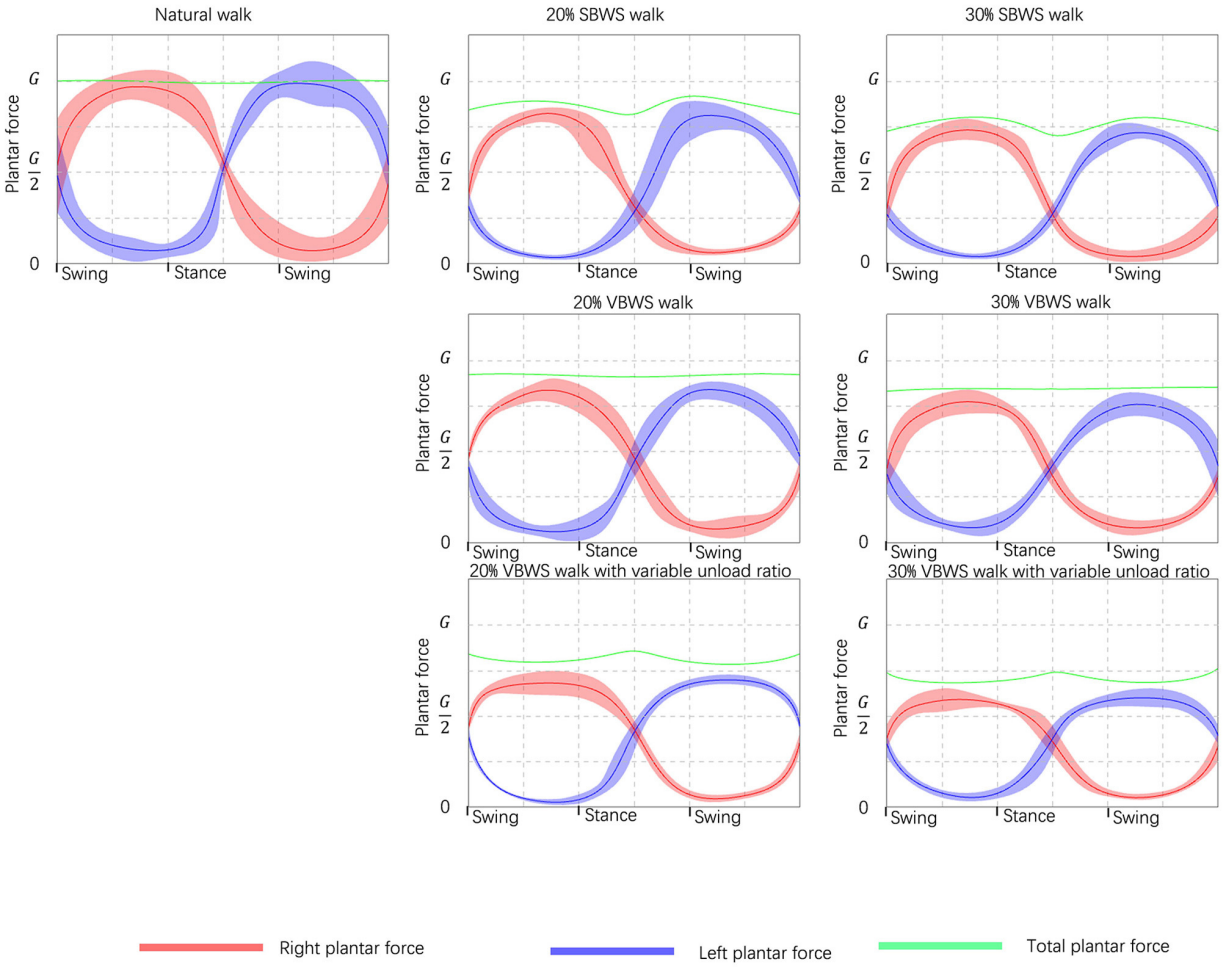
Comparison of the BWS effects under different unload modes. Taking the force data read from plantar pressure sensors as validation references, from left to right it shows the increasing weight supporting force, and from top to bottom it shows the different unload modes.

Figure 10 illustrates the changes in plantar pressure relative to the gait phase for five subjects walking without any BWS assistance (natural walk) and walking with 20 and 30% BWS force under the SBWS walk mode, FUR-VBWS walk mode, and VUR-VBWS walk mode. Since the weight of each subject was different, we unified the experimental results by using the BWS ratio as a reference, which facilitated the comparison of multiple subjects' experimental results simultaneously. We used plantar pressure as a percentage of body weight $F_{GRFr}^{i}$. The data were processed as follows:


(56) $$\begin{array}{l} F_{GRFr}^{i}=\frac{F_{GRF}^{i}}{G},i\;\in \left\{l,\;r\right\} \end{array}$$

Additionally, Figure 10 illustrates the mean plantar pressure ratio data and margin of error for the 15-step gait experiment in five subjects. During natural walking without BWS, the plantar pressures of the left and right feet sequentially reached their peak and trough with the gait phase, and the legs alternated to support the body weight to complete a gait cycle, and the total pressure of the left and right feet is approximately equal to G.

In the SBWS walking experiment, the maximum sum of plantar pressures of both feet occurred on average at 35 and 77% of each gait cycle, in the middle of the swing phase. This value was significantly larger than the sum of plantar pressures of both feet in the stance phase, and the left and right legs supported the weight separately at this time. This was due to the subject's body COM being highest during the single-leg support phase compared to the weight-support mechanism, even if a fixed weight-support value was set. The minimum sum of plantar pressure of both feet occurred in the middle of the stance phase at 51% of each gait cycle on average, consistent with the set BWS of 20%, and the subject's left and right legs jointly supported the weight at this time.

In the FUR-VBWS walking experiment, the total sum of plantar pressures of both feet remained nearly constant throughout the gait cycle, as the weight-support system followed the body's COM to move in the Z-axis direction. However, the left and right feet still needed to bear the weight separately in the swing phase, resulting in more obvious fluctuations in the plantar pressure value of one foot. The plantar pressure of one foot was also different under the 20 and 30% weight-support ratio.

In the VUR-VBWS walking experiment, the system doubled the weight-support ratio in the swing phase of single-leg support, resulting in less fluctuation in the plantar pressure on the left and right foot in one gait cycle. The average error in different gait phases of plantar pressure for each foot was 0.22 and 0.39 G for 20 and 30% weight-support ratio, respectively. This met our expectation for the VUR-VBWS experiment of the walker, which demonstrated that, compared to SBWS and conventional VBWS systems, our proposed VUR-VBWS system could significantly reduce fluctuations in BWS ratios during the transition between different gait events.

The data from the above experiments are presented in Table 2.

**Table 2:** Plantar pressure data under different unloading force and assistance modes are read and calculated from the sensors.

**BWS strength**	**Sensor**	**SBWS walk swing**	**SBWS walk stance**	**VBWS walk swing**	**VBWS walk stance**	**VUR-VBWS walk swing**	**VUR-VBWS walk stance**
20% unloading force	Left plantar pressure	0.88 ± 0.03G	0.41 ± 0.03G	0.86 ± 0.04G	0.42 ± 0.02G	0.65 ± 0.03G	0.46 ± 0.02G
	Right plantar pressure	0.9 ± 0.07G	0.39 ± 0.01G	0.86 ± 0.03G	0.43 ± 0.01G	0.66 ± 0.01G	0.44 ± 0.01G
	Total plantar pressure	0.92G	0.78G	0.88G	0.87G	0.67G	0.91G
30% unloading force	Left plantar pressure	0.78 ± 0.03G	0.38 ± 0.02G	0.75 ± 0.03G	0.38 ± 0.02G	0.53 ± 0.02G	0.44 ± 0.02G
	Right plantar pressure	0.77 ± 0.03G	0.37 ± 0.01G	0.76 ± 0.03G	0.37 ± 0.01G	0.54 ± 0.02G	0.43 ± 0.01G
	Total plantar pressure	0.81G	0.75G	0.77G	0.76G	0.55G	0.88G

#### 3.2.2. Feasibility of MT

**Figure 11:**
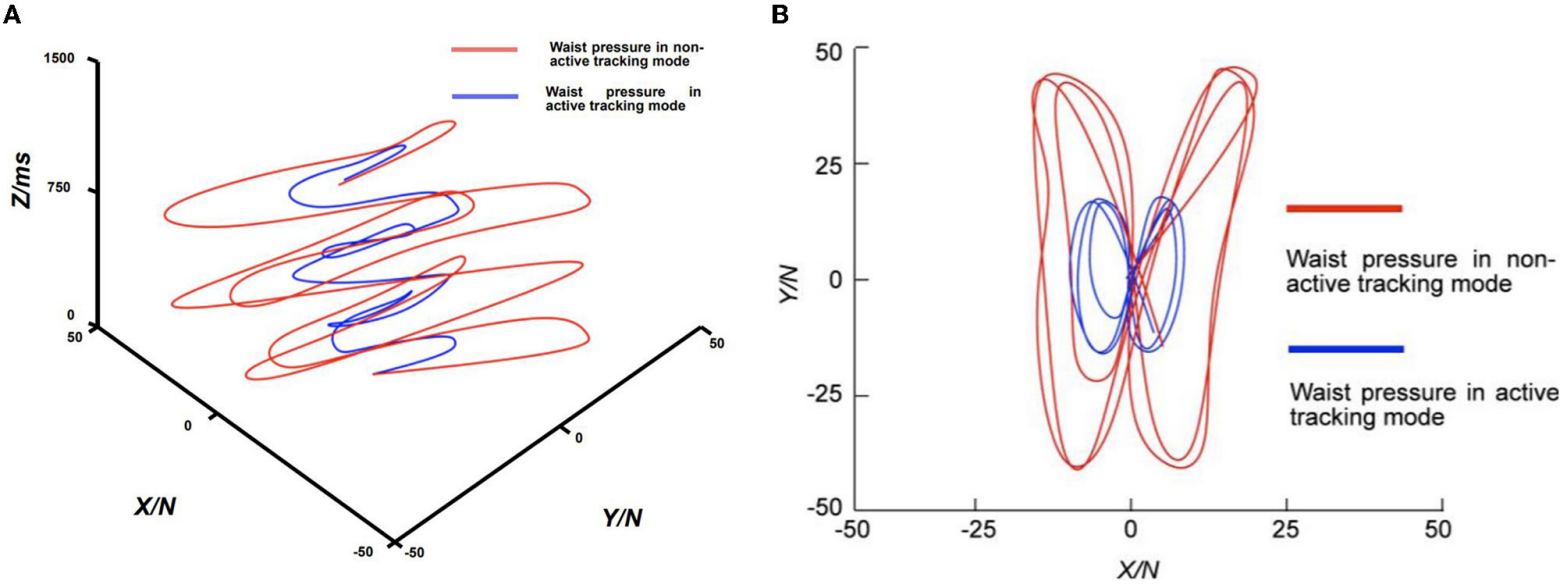
Comparison of waist pressure curves with active tracking mode enabled and disabled. **(A)** Perspective view of pressure against time; **(B)** Top view of pressure in XY directions.

Figure 11 shows the force on the waist in the horizontal direction for the five subjects performing 15 steps of rhythmic walking with MT mode off and on, respectively. The walker was connected to the subjects through the harness, so we can assume that this force reflects the degree of obstruction of the walker to the subjects during the walking process.

In Figure 11A, we illustrate the changes in the subject's waist plane forces in the X and Y directions (N) relative to time (milliseconds) during the walking training experiment under the MT mode, as collected and analyzed. In the figure, the X and Y axes represent the forces experienced by the subject in the corresponding X and Y directions, while the Z-axis represents the corresponding time. We can see that the horizontal force on the waist of the patient with the MT function on is much smaller than the horizontal force on the waist of the patient with the MT function off during the entire course of the 1,500 ms follow-walk experiment. In Figure 11B, the average waist forces during walking were 9.4 N in the X-direction and 23.5 N in the Y-direction when the MT function was turned off, while the average forces were 4.3 N and 8.2 N when the MT function was turned on, respectively. A 76.4% reduction in the overall waist force was achieved when the MT function was enabled compared to when it was disabled. This result indicates that the MT system can effectively reduce the horizontal dragging effect of the walker on the patient, thereby making the direction of the BWS force more aligned with the vertical direction. While this suggests the potential for an improved user experience, further investigation with user feedback is necessary to confirm this enhancement.

#### 3.2.3. System safety validation

In the control system, we set the MT mode such that the fastest system movement speeds for the walker of 1,000 mm/s along the Y-axis and 500 mm/s along the X-axis were achieved under the force of 25 N for both axes, as shown in Table 3. In the maximum movement speed test experiment, the encoder feedback indicated that the maximum speeds reached were 1,035 ± 65 mm/s for the Y-axis and 541 ± 38 mm/s for the X-axis, with corresponding forces of 211 ± 56 N and 165 ± 32 N, respectively. This implies that when pushing the assistive device system in any direction on a plane with a force exceeding the maximum input control system force, the maximum travel speed is still limited to within 110% of the maximum speed.

**Table 3:** Safety test on the max speed and brake distances in multiple directions under extreme situations.

**Mode**		**Max ^@^ Y-axis (forward)**	Max ^@^ X-axis (both wards)^*^	**Endpoint ^@^ Y-axis (forward)**	Endpoint ^@^ X-axis (both wards)^*^	**Max ^@^ Z-axis (downward)**
MT	Speed (mm/s)	1,035 ± 8	538 ± 11	1,021 ± 9	522 ± 14	N/A
	Corresponding force (*N*)	211 ± 56	165 ± 32	195 ± 58	162 ± 33	N/A
	Distance (mm)	N/A	N/A	52 ± 11	36 ± 7	N/A
VBWS	Distance (mm)	N/A	N/A	N/A	N/A	23 ± 8
	Corresponding force (*N*)	N/A	N/A	N/A	N/A	514 ± 156

^*^The maximum value occurring among the two directions.

In the horizontal instantaneous stop sliding distance test, the measured sliding distances were 52 ± 11 mm along the Y-axis and 36 ± 7 mm along the X-axis, indicating that the system responded relatively quickly to the emergency stop command and halted its movement. In the longitudinal instantaneous stop sliding distance test, as previously mentioned, the VBWS system was set to the “Normal BWS Value,” with values exceeding this threshold deemed indicative of a falling tendency, which immediately stops the descent of the harness module. In the test, the force applied far exceeded the “Normal BWS Value,” triggering the VBWS mode to halt and measuring a downward displacement of 23 ± 8 mm for the harness module along the Z-axis. The full-force squat measured an instantaneous force of 514 ± 156 N. Throughout the testing process, no noticeable slippage of wheels or mechanical moving components was observed, except during the emergency stop distance test.

#### 3.2.4. Limitations

By evaluating the design features and experimental data, we identified several limitations of the study in the system design and experimental process. First, the force sensors used for intention recognition in the system design require a sequence of events: user generates intention, initiates movement, force reaches a certain threshold to trigger force sensor recognition, recognition signal is sent to the control system, and the actuator is driven to move. The delays from the sensors, control system, and driving and actuating systems cannot be ignored, and the accumulated delay results in noticeable fluctuations in weight reduction ratio and horizontal dragging for the user. Second, for patients with lower limb mobility impairments caused by conditions such as osteopathy or stroke, their movements may not fully correspond to their intentions due to insufficient support or muscle spasms. Even when the patient is unable to maintain balance under the VBWS system, the MT system follows the patient entirely, unable to provide necessary horizontal support. Finally, due to experimental conditions, no actual patients participated in the testing process, and the demographics of the subjects in this study were quite uniform, as this was a preliminary investigation. Consequently, the generalizability of our findings to a more diverse subject population may be limited, and our discussion of the experimental results is limited to the effects presented by the data from the machine itself, such as reduced dragging effects and smaller VBWS fluctuations. The impact on patients requires further evaluation through clinical trials centered on obtaining physiological data from patients.

To address the first two issues, our next steps involve obtaining the movement intentions of patients during the training process in real-time, swiftly and accurately, allowing us to bypass the sensor-sensing step and directly drive the MTVBWS system through intention signals, potentially significantly reducing latency. Additionally, with precise intention recognition, movements unrelated to the patients' intentions will not trigger system actions, potentially enhancing the support provided by the assistive device system and increasing rehabilitation efficiency. Regarding the final issue, after optimizing the system design and experimental process, we plan to involve participants with a broader range of ages, genders, and physical conditions to better evaluate the performance and safety of the walker system across different individuals, and collaborate with a hospital rehabilitation center to provide suitable patient participants while ensuring proper rehabilitation training services and patient safety during the training process.

## 4. Conclusion

In this article, we introduce a walker with VBWS that actively tracks a subject, aiming to safely assist patients with lower limb mobility impairments due to mid- to late-stage stroke during ground-based walking rehabilitation training. And we conducted experiments to verify the advantages of these functions and the safety assurance design.

For VUR-VBWS effect validation, we used foot pressure sensors to collect plantar pressure data and time as indicators to evaluate real-time BWS. The experimental design compared SBWS, VBWS, and VUR-BWS modes, verifying the latter's advantage in BWS stability. Our experiments showed that, compared to the SBWS mode, the VBWS mode yields a smaller average force difference between the Swing and Stance phases for both feet, proving that the VBWS system can further reduce overall plantar pressure force fluctuations during gait event transitions. The VUR-VBWS mode, which adjusts BWS ratios based on gait event judgment, further reduced the average pressure difference between the Swing and Stance phases for each foot, demonstrating a relatively constant BWS ratio for each foot and lower limb during different gait events.

Regarding MT effect validation, we employed a 3D force sensor at the waist to collect horizontal force data and time as indicators to evaluate the system's ability to mitigate horizontal dragging effects. Analysis of the force situation along the X and Y axes during 15 steps of walking with MT mode turned off and on revealed a significant reduction in the average force experienced by the user, indicating smaller horizontal dragging resistance.

In safety assurance testing, we conducted extreme usage scenario tests to verify the system's fault tolerance in response to emergency user inputs, unexpected user actions, and environmental obstacles. The experimental data show that the system can provide a certain degree of protection for user safety, to some extent, against loss of control caused by the user or the system itself, as well as falls.

In our future work, we aim to improve the performance of our system while ensuring the stability and safety of both the control system and mechanical structure. Specifically, we plan to reduce the system delays during walking training by improving the system's response to user inputs. Additionally, we plan to implement different reduction levels of force fluctuations while walking, which can be set to accommodate different levels of lower-limb impairments, or gradually increase the intensity of the training for bearing the force fluctuations. To validate our approach, collaborating with local doctors and professionals in the field, we will conduct further experiments with both able participants and patients of different gender, age, height, and weight.

## Data availability statement

The original contributions presented in the study are included in the article/supplementary material, further inquiries can be directed to the corresponding authors.

## Ethics statement

The studies involving human participants were reviewed and approved by Institutional Review Board of Shenzhen Institute of Advanced Technology, Chinese Academy of Sciences. The patients/participants provided their written informed consent to participate in this study. Written informed consent was obtained from the individual(s) for the publication of any potentially identifiable images or data included in this article.

## Author contributions

PS, XZ, and BL: conceptualization, validation, supervision, project administration, and funding acquisition. PS and XZ: methodology. XZ: software, formal analysis, investigation, resources, data curation, writing—original draft preparation, writing—review and editing, and visualization. All authors agree to be accountable for the content of the work.
